# Comparison of total patellectomy and osteosynthesis with tension band wiring in patients with highly comminuted patella fractures: a 10–20-year follow-up study

**DOI:** 10.1186/s13018-021-02656-3

**Published:** 2021-08-13

**Authors:** Xiangtian Deng, Lian Zhu, Hongzhi Hu, Jian Zhu, Weijian Liu, Junzhe Zhang, Sifan Yang, Zhipeng Ye, Haitao Guan, Boyu Zhang, Xiaodong Cheng, Yingze Zhang

**Affiliations:** 1 School of Medicine, Nankai University, Tianjin, 300071 People’s Republic of China; 2 Department of Orthopaedic Surgery of Hebei Province, Third Hospital of Hebei Medical University, 139 Ziqiang Road, Shijiazhuang, 050051 Hebei People’s Republic of China; 3 NHC Key Laboratory of Intelligent Orthopaedic Equipment, Third Hospital of Hebei Medical University, Shijiazhuang, 050051 Hebei People’s Republic of China; 4 Department of Orthopedics, Union Hospital of Tongji Medical College of Huazhong University of Science and Technology, Wuhan, 430022 People’s Republic of China

**Keywords:** Patella comminuted fractures, Total patellectomy, Open reduction and internal fixation, tension band wiring, Outcomes

## Abstract

**Background:**

The purpose of this study was to evaluate and compare the long-term clinical outcomes between the total patellectomy and osteosynthesis with tension band wiring in patients with highly comminuted patella fractures.

**Methods:**

Between January 1987 and December 2003, this retrospective study included a total of 35 patients (mean age, 51.4±16.8 years) with a minimum of 10 years follow-up period, comprising 29 males and 6 females, who were divided into the total patellectomy group (17 patients) or the open reduction and internal fixation (ORIF) group (18 patients) in the Third Affiliated Hospital of Hebei Medical University. We retrospectively collected patient demographics and data on the type of trauma, fracture type, and postoperative complications. Clinical outcomes including knee range of motion (ROM), 36-Item Short-Form Health Survey (SF-36) score [including physical component score (PCS) and mental component score (MCS)], Knee Injury and Osteoarthritis Outcome Score (KOOS), and Kujala score were evaluated and compared between the two groups. Biodex System dynamometer was used to quantitatively evaluate quadriceps femoris muscle power following measurement of peak torque.

**Results:**

The mean follow-up periods of the total patellectomy group and the ORIF group were 17.2±5.6 and 16.8±4.9 years, respectively. There were no significant differences between the two groups of patient demographics in terms of the number of patients, age, sex, injury side, time to surgery, type of trauma, and fracture classification (*p*>0.05). Total patellectomy was comparable to osteosynthesis with tension band wiring in terms of ROM [injured knee: 120.4±3.1° *vs* 118.6±3.3°; uninjured knee: 126.5±2.8° *vs* 127.3±1.7°; both *p*>0.05], peak torque [Injured knee: 96.2±2.3 *vs* 97.3±2.6, N· m; Uninjured knee: 107.6±2.1 *vs* 106.3±1.8, N· m; both *p*>0.05], SF-36 score [PCS: 64.1±18.0 *vs* 61.5±17.9; MCS: 55.1±13.8 *vs* 54.3±12.4; both *p*>0.05], KOOS score [76.3±12.1 *vs* 73.4±11.7; *p*>0.05], and Kujala score [67.6±11.8 *vs* 70.8±11.9; *p*>0.05] at the final follow-up, while total patellectomy had significantly shorter operation time than ORIF group (47.5±12.1 *vs* 68.8±22.3, min, *p*<0.05). In the total patellectomy group, complications occurred in 6 of 17 cases (35.3%), and all occurred with calcification. In the ORIF group, complications occurred in 12 of 18 cases (66.7%), including 2 cases of infection (11.1%), 1 case of non-union (5.6%), 2 cases of implant failure (11.1%), 2 cases of soft tissue irritation (11.1%), and 5 cases of patellofemoral arthritis (27.8%).

**Conclusions:**

Total patellectomy technique was a safe and reliable alternative treatment for treating patients with highly comminuted patella fractures when anatomically reduction and rigid fixation were difficult, although it caused relatively higher rates of calcification.

## Background

Patella is situated between the quadriceps femoris muscle tendon and the patella tendon; it is an important component of the knee extension system with increasing the moment arm of the quadriceps extensor mechanism [1, 2]. Patella fractures, accounting for about 3.5% of lower extremities fractures [3], mainly result from high-energy trauma and can result in stiffness, patellofemoral arthritis, and disabling sequelae if not treated appropriately. Comminuted fractures of the patella comprise about 55% of operatively managed patellar fractures [4], and their treatment depends on the comminution of fracture fragments. Patients with highly comminuted patella fractures often lead to disrupted extensor mechanisms and considerable functional disability. Although numerous studies have emphasized the importance on internal fixation of patella comminuted fractures, the optimal surgical management remains controversial. Furthermore, it is undeniable that open reduction and internal fixation (ORIF) have many postoperative complications, including infection, limited range of motion, post-traumatic osteoarthritis, delayed union or nonunion, soft-tissue irritation, and implant-associated problems [5, 6]. Overall, surgeons were required to restore the quadriceps extensor mechanism, maintain the articular congruity, and provide stable fixation for early rehabilitation. However, it remains difficult and technically demanding to achieve anatomical reduction and stable fixation in patients with comminuted patella fractures as they are usually along with highly small stellate fragments, and total patellectomy is sometimes inevitable when reduction is felt to be futile.

The appropriate surgical intervention to obtain a favorable functional outcome in comminuted patella fractures has been a topic under debate for decades [7, 8]. In clinical practice, however, we have found that it is difficult to obtain sufficient stability with highly comminuted patella fractures. Patellectomy is a relatively old surgical modality, including partial and total patellectomy, and has been demonstrated to exert satisfactory clinical outcomes when precise anatomical reduction cannot be achieved by internal fixation [9, 10]. However, Wilkinson et al. [11] have shown that comminuted patella fractures operatively treated by total patellectomy would lead to a high incidence of subjective dissatisfaction, detrimental effect on quadriceps power, and poor functional outcome. Carpenter et al. [12] identified that total patellectomy results in approximately 50% reduction in quadriceps strength. Hence, the long-term clinical outcome of total patellectomy on knee function has been a matter of controversy.

This study retrospectively compared the clinical outcomes of total patellectomy and ORIF with tension band wiring in treating highly comminuted patella fractures. Patients treated with total patellectomy were defined as the total patellectomy group, and those who were treated with tension banding wiring were defined as the ORIF group. We compared two groups regarding operation time, range of motion, knee function scores, and postoperative complications from the 10–20-year follow-up period. The functional scoring system included the 36-item Short-Form Health Survey (SF-36) score [consisting of physical component score (PCS) and mental component score (MCS)], the Knee Injury and Osteoarthritis Outcome Score (KOOS), and the Kujala scoring system. Quadriceps femoris muscle power was quantitatively assessed by the Biodex System dynamometer in measuring peak torque.

Until now, few literatures on total patellectomy have focused on highly comminuted patella fractures nor did they compare total patellectomy to ORIF with tension band wiring on long-term clinical outcomes. Therefore, the purpose of the present study was to (i) comprehensively evaluate the long-term stages of clinical results of total patellectomy and ORIF with tension banding wiring, (ii) compare the postoperative knee function at the long-term follow-up period using the two surgical techniques, and (iii) evaluate the incidence of complications regarding the total patellectomy and ORIF with tension band wiring. The hypothesis was that patients treated by total patellectomy would have similar clinical outcomes with lower incidence rates of complication than those treated by osteosynthesis with tension band wiring.

## Materials and methods

### Ethics statement

This retrospective comparative study was performed at the Third Hospital of Hebei Medical University and approved by the local institutional ethics committee. All enrolled patients provided written informed consent and this study complied with the principles of the Declaration of Helsinki.

### Patients

**Fig. 1:**
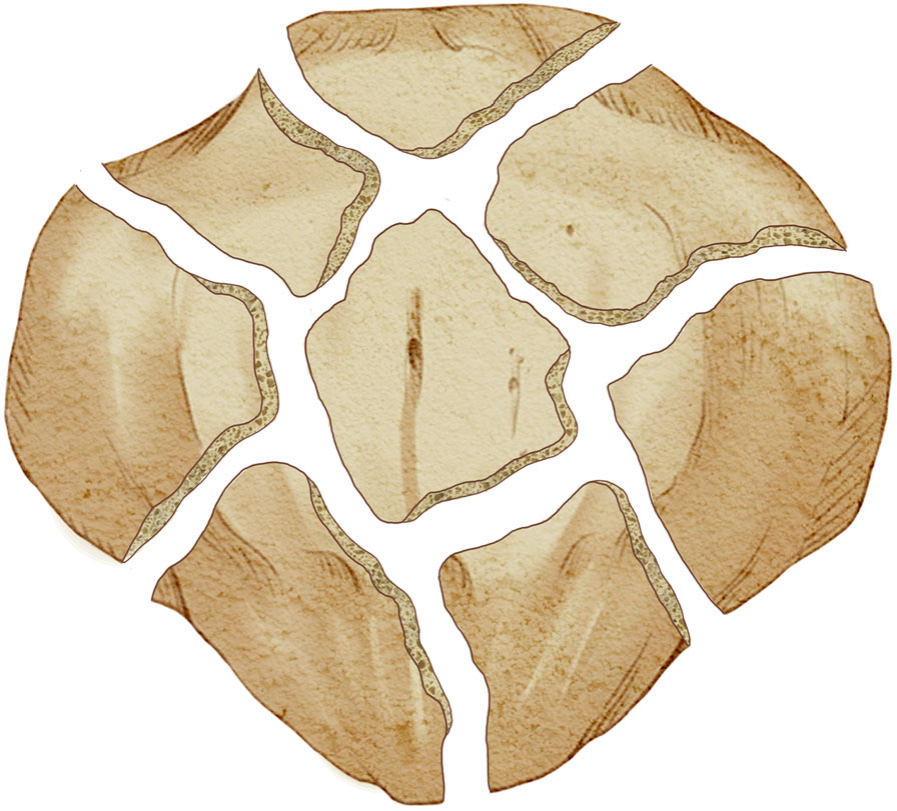
Schematic illustration of the highly comminuted patella fractures

Between January 1987 and December 2003, all patients who were admitted to our department for the treatment of patella fractures were collected. Inclusion criteria for this present study were as follows: ① aged older than 18 years, ② unilaterally isolated highly comminuted patella fractures, ③ patients treated either by total patellectomy or ORIF with tension band wiring, ④ the postoperative functional scores and complication rates were considered as the comparison, ⑤ patients with complete follow-up outcomes and with a minimum follow-up time period more than 10 years, and ⑥ the study was designed as a retrospective comparative study. Highly comminuted fractures were defined as multi-fragmentary fractures with massive comminution or diastasis fragments, such as fracture fragments of more than 4 parts of the body of the patella and/or a separation of fragments more than 3 mm [5], as illustrated in Fig. 1.

Patients were excluded if any of the following were present: (a) pre-existing limited range of motion of the knee, (b) open fractures and/or pathological fractures, (c) a history of previous knee surgery, (d) fractures older than 21 days, (e) displaced avulsion fractures of the inferior or superior patella pole, and (f) patients were lost to follow-up. All cases underwent surgical treatment by the same senior orthopedic surgeon (Y.Z.Z) of our institution.

A total of 35 patients with a minimum of 10 years of follow-up were included in this study and assigned into the total patellectomy group (*n*=17) and ORIF group (*n*=18), respectively. Patient demographic characteristics included age at the time of operation, sex, injury side, type of trauma, time to surgery, fracture classification, and follow-up time were recorded, as presented in Table 1. The definition of follow-up time was determined by the time of operation date to the time of the last outpatient visit. Fracture classification of the patella comminuted fracture segments was recorded on the first day of admission according to the Regazzoni classification system [13], by reviewing the pre-operative radiographs. Regazzoni classification included three fracture patterns: type A (longitudinal fractures), type B (transverse fractures), and type C (comminuted fractures) based on the fracture morphology. In addition, type C was further categorized into three subgroups. Type C1 was defined as non-displaced fractures, type C2 was defined as displaced fractures with separation of fragments less than 2 mm, while type C3 represented comminuted fracture with separation of fragments more than 2 mm.

**Table 1:** Patient demographic characteristics

Variables	Total patellectomy group	ORIF group	*p* value
Number of patients	17	18	-
Age (years)	50.6±17.1	52.1±16.3	0.792
Sex			0.628
Male	15	14	
Female	2	4	
Side			0.631
Left	10	12	
Right	7	6	
Time to surgery (days)	3.6±2.7	4.1±3.5	0.545
Type of trauma			0.876
Traffic accident	8	9	
Fall from height	6	5	
Direct blow	3	4	
Regazzoni classification			0.581
Type C1	0	0	
Type C2	3	2	
Type C3	14	16	
Follow-up time (years)	17.2±5.6	16.8±4.9	0.823

The values are presented as the mean±SD or *n*

### Surgical technique

**Fig. 2:**
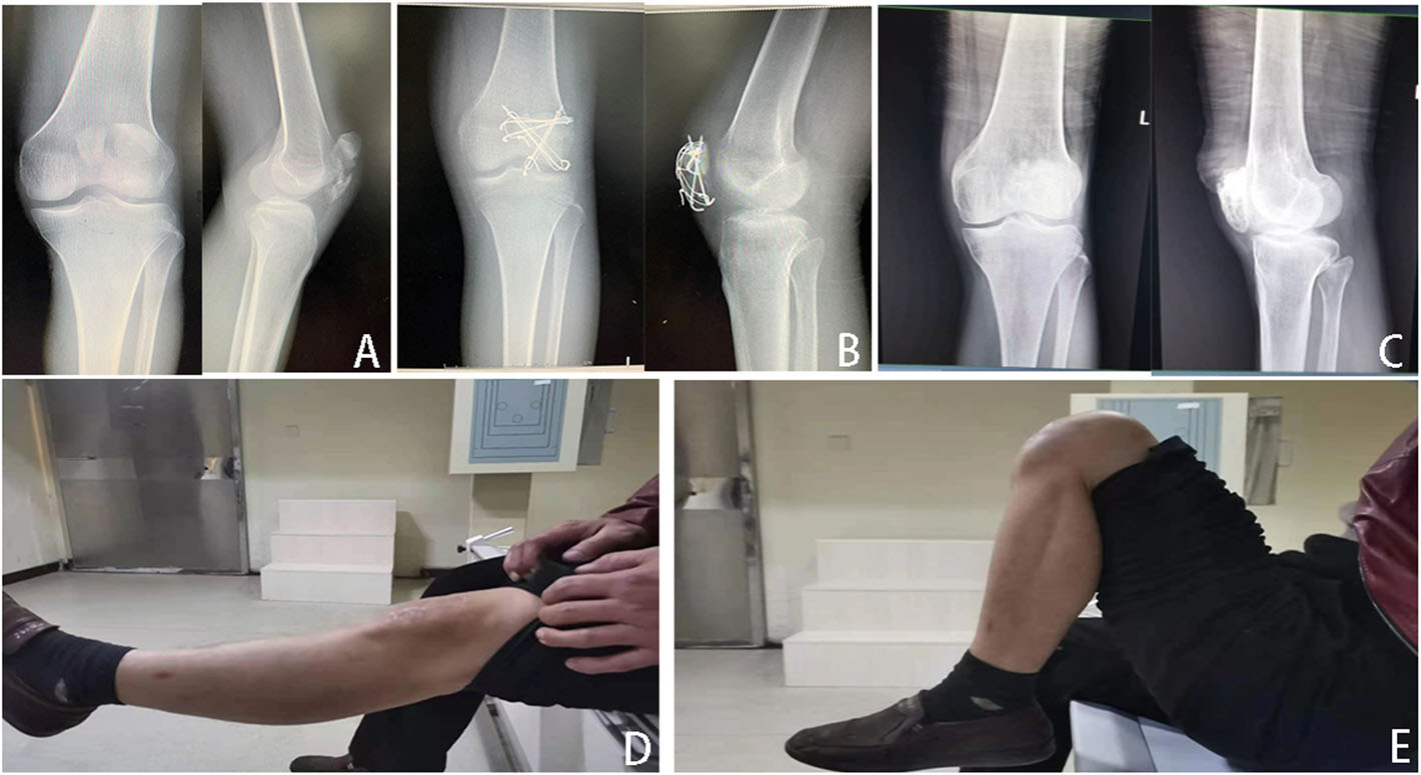
**A** Male, 41 years old, traffic accident, anteroposterior and lateral radiographs of the left knee showed patellar comminuted fractures preoperatively and **B** postoperatively at 1 year showed the fracture was well healed. **C** Anteroposterior and lateral radiographs of the left knee postoperatively at the final follow-up showed patellofemoral arthritis. **D** Range of motion was restored normally at the final follow-up

**Fig. 3:**
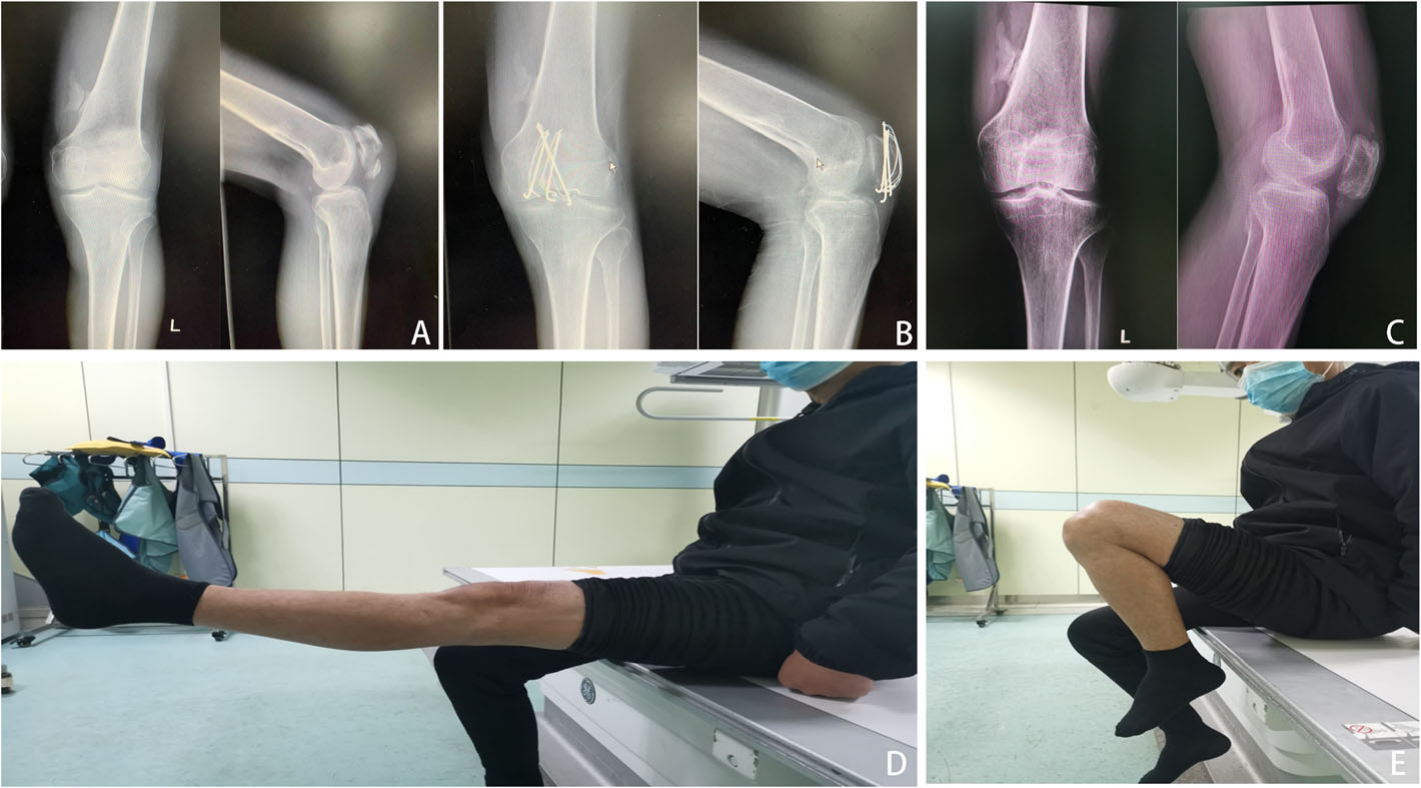
**A** Male, 58 years old, traffic accident, anteroposterior and lateral radiographs of the left knee showed patellar comminuted fractures preoperatively and **B** postoperatively at 1-year showed fracture healing. **C** Anteroposterior and lateral radiographs of the left knee postoperatively at the final follow-up showed patellofemoral arthritis. **D** Range of motion was restored normally at the final follow-up

**Fig. 4:**
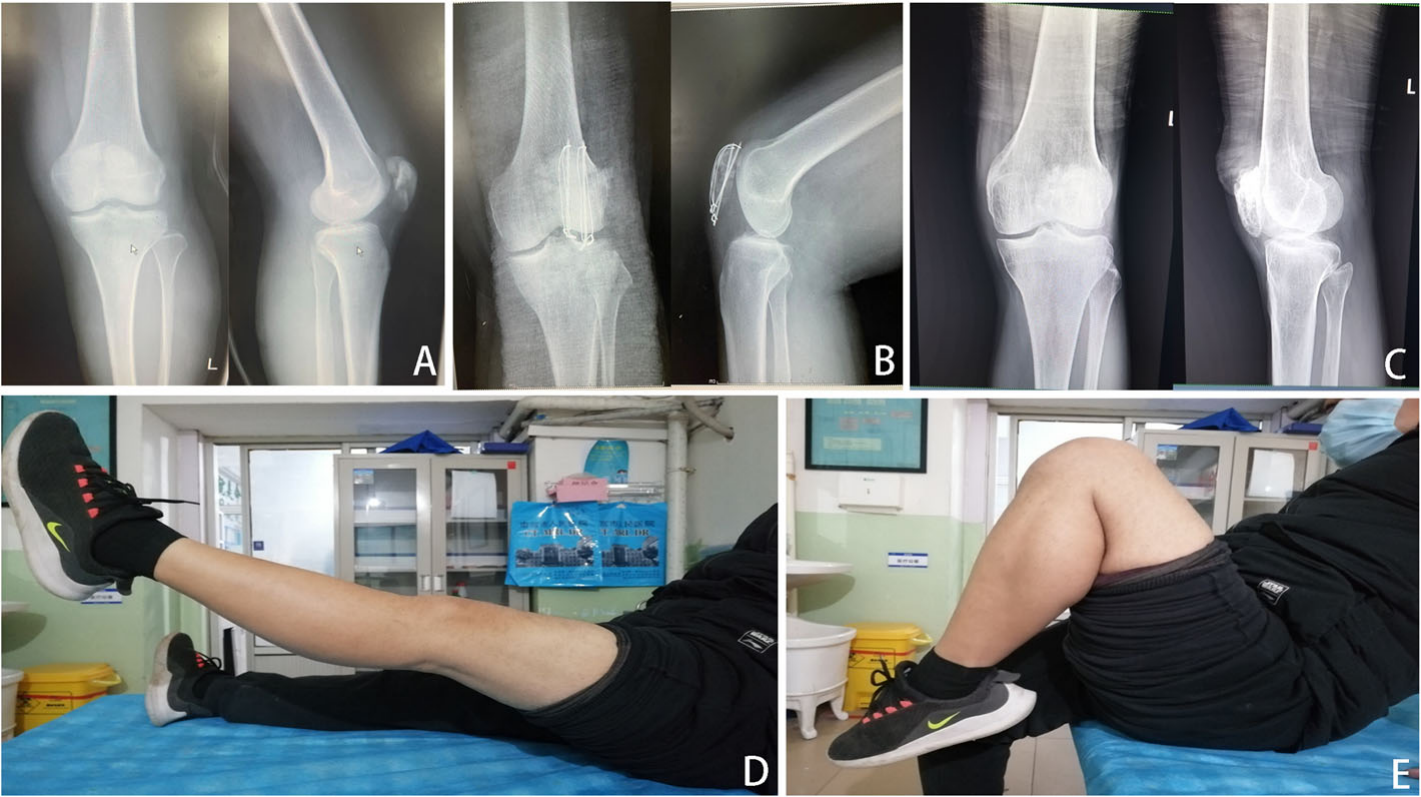
**A** Male, 45 years old, direct blow, anteroposterior and lateral radiographs of the left knee showed patellar comminuted fractures preoperatively and **B** postoperatively at 1 year, the fracture was well healed. **C** Anteroposterior and lateral radiographs of the left knee postoperatively at the final follow-up showed patellofemoral arthritis. **D** Range of motion was restored normally at the final follow-up

**Fig. 5:**
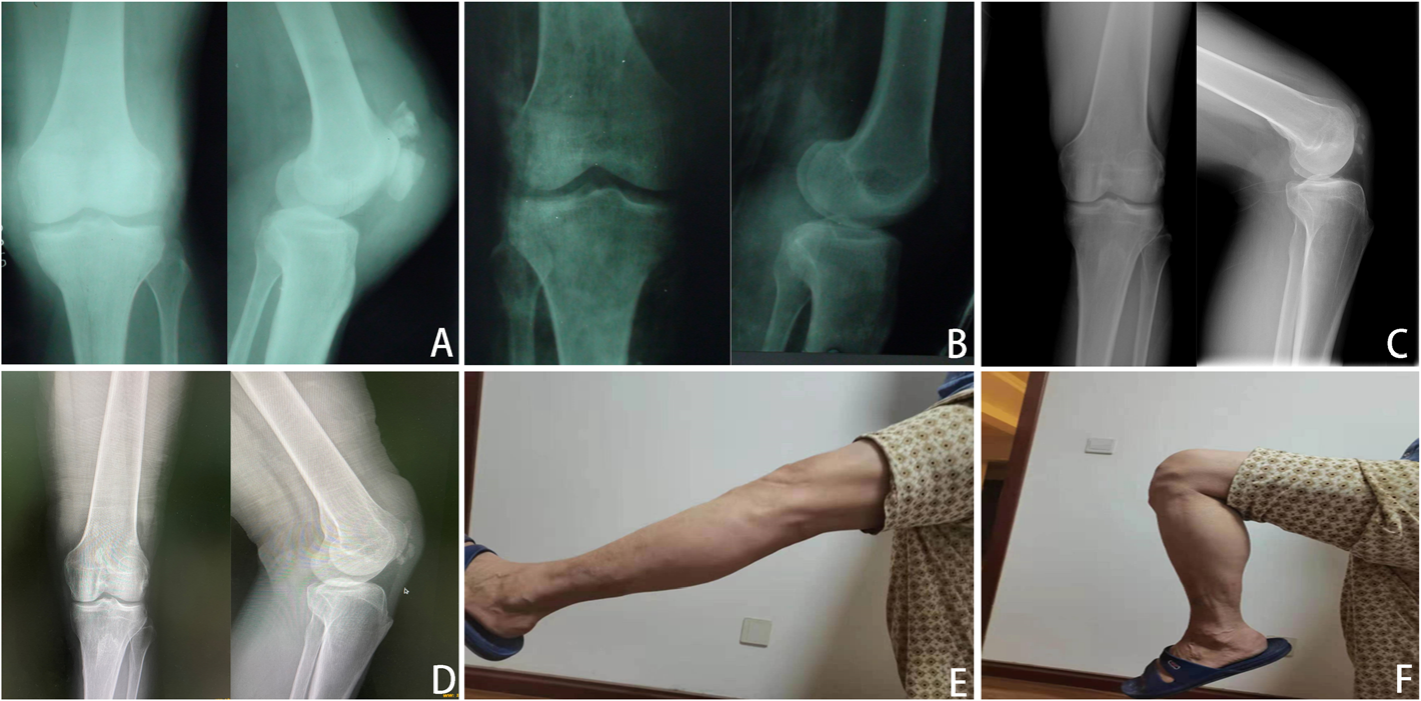
**A** Male, 57 years old, traffic accident, anteroposterior and lateral radiographs of the left knee showed patellar comminuted fractures preoperatively and **B** postoperative immediately, **C** postoperatively at 10 years with calcification developed. **D** Anteroposterior and lateral radiographs of the left knee postoperatively at the final follow-up. **E** Satisfactory functional outcome was restored normally at the final follow-up

**Fig. 6:**
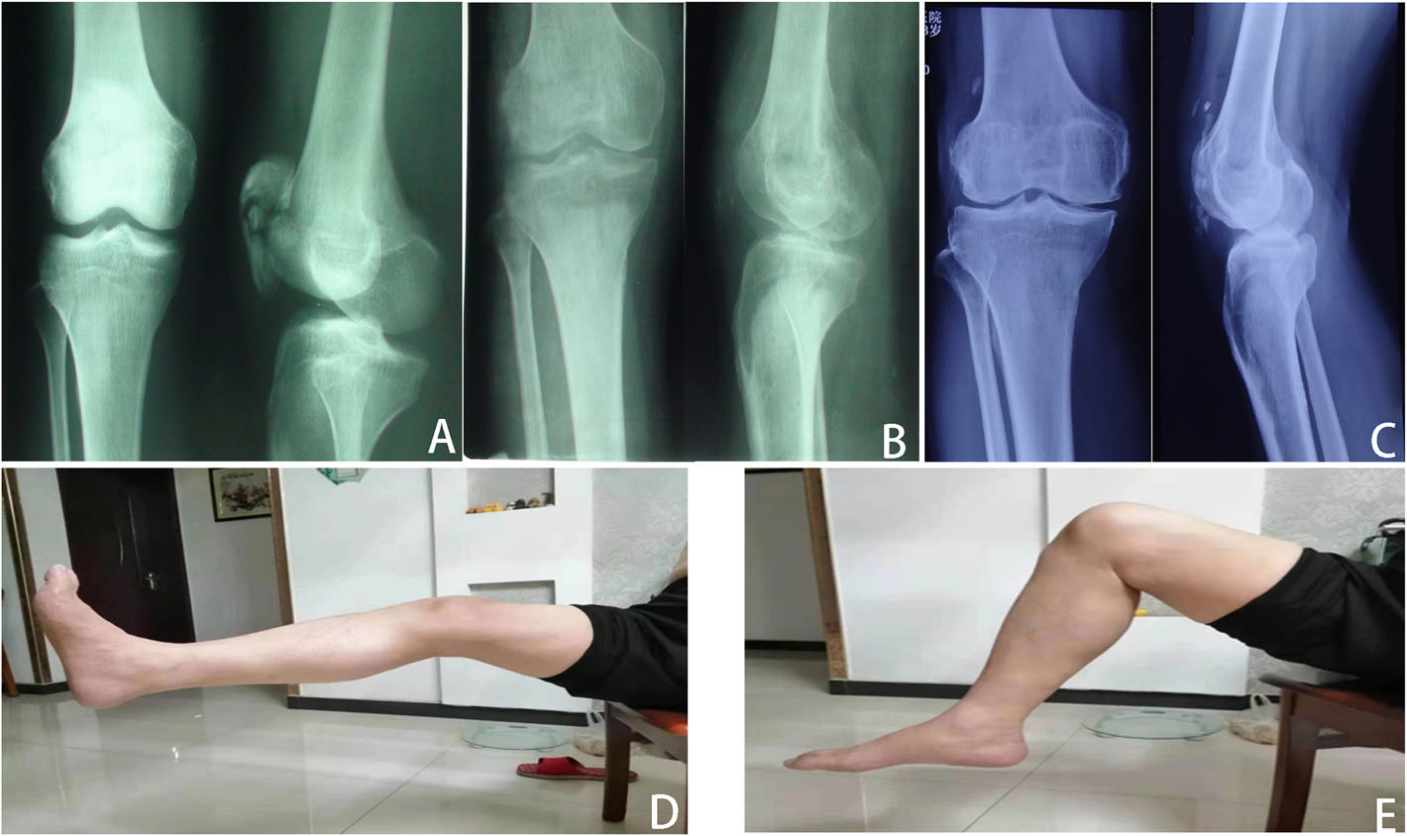
**A** Male, 52 years old, fall from height, anteroposterior and lateral radiographs of the right knee showed patellar comminuted fractures preoperatively and **B** postoperative immediately after surgery. **C** Anteroposterior and lateral radiographs of the left knee postoperatively at the final follow-up. **D**, **E** Satisfactory functional outcome was restored normally at the final follow-up

**Fig. 7:**
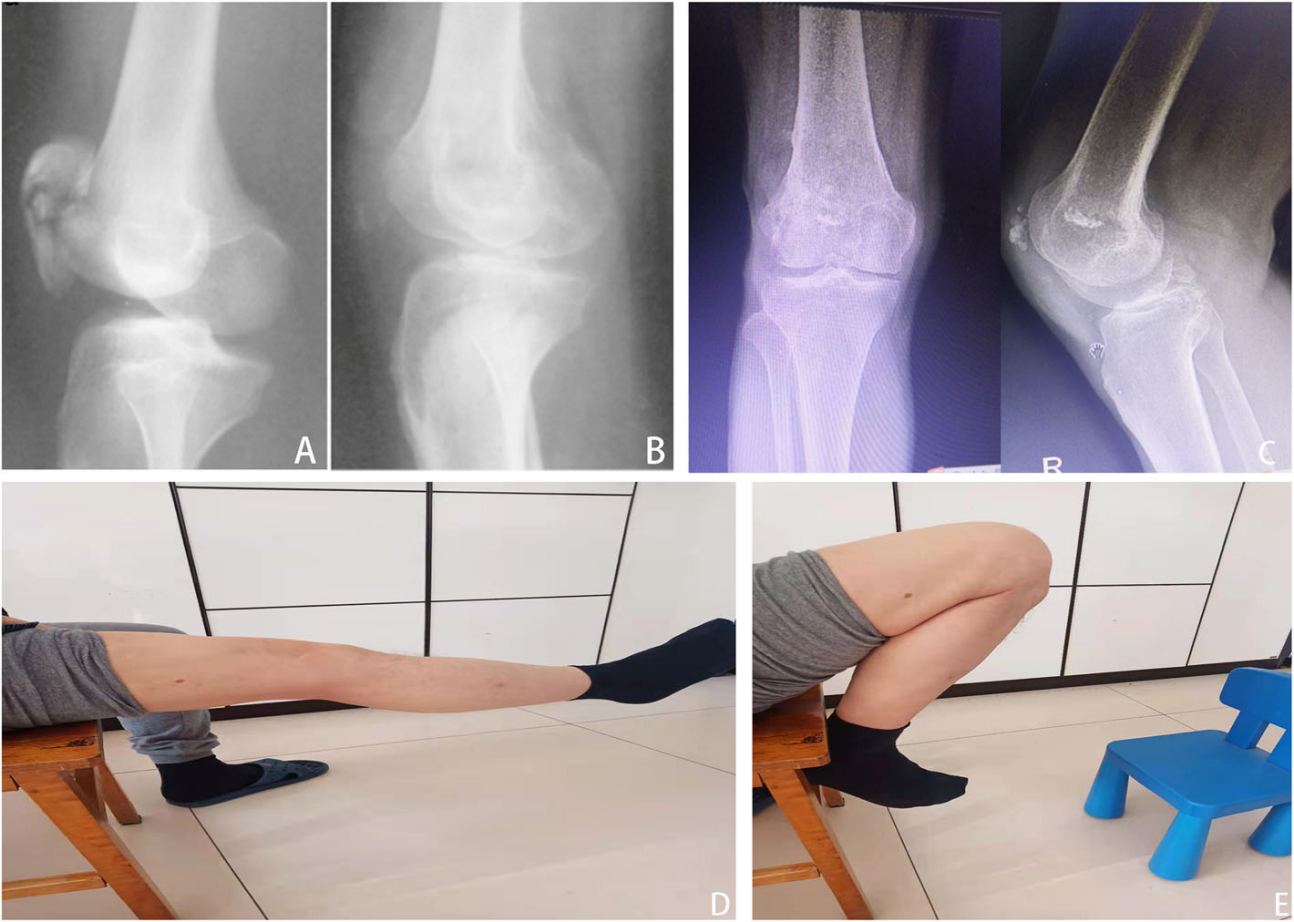
**A** Male, 56 years old, traffic accident, lateral radiographs of the right knee showed patellar comminuted fractures preoperatively and **B** postoperative immediately after surgery. **C** Anteroposterior and lateral radiographs of the left knee postoperatively at the final follow-up. **D** and **E** Satisfactory functional outcome was restored normally at the final follow-up

All patients were managed with spinal anesthesia and placed in a supine position with 20° flexion of the knee on a radiolucent table, and a pneumatic tourniquet was applied in the proximal thigh. The surgical modality was determined at the preference of the surgeon with the purpose of anatomical reduction of patella fractures whenever possible. In the ORIF group**,** we performed a straight midline skin incision (about 8–10 cm length) extending from the upper pole of the patella to the tibial tuberosity, and the fracture fragments were completely exposed and intra-articular hematomas were debrided. After that, the fracture was reduced by the use of reposition forceps, and a tension band with Kirchner wires was performed to fixed fracture fragments under direct visualization. The stability of the fracture was examined through a full range of flexion and extension intraoperatively. Instead, the total patellectomy we performed is similar to that described by Wilkinson et al. [11]. Typical cases with tension band wiring were shown in Figs. 2, 3, and 4. Total patellectomy was only conducted when reduction is felt to be difficult. Typical cases of total patellectomy at the final follow-up were shown in Figs. 5, 6, and 7.

### Postoperative rehabilitation

Postoperative rehabilitation protocol was performed individually depending on the treatment of surgical technique. Patients who were treated by the tension band wiring techniques started to perform active flexion exercises of the knee and were instructed to perform quadriceps femoris contraction exercises after the operation*.* At 3 weeks postoperatively, patients were progressed with active extension exercises training. At 6 weeks postoperatively, full weightbearing was allowed during level walking.

Patients who were treated by total patellectomy had a hinged knee brace or long leg cast at full extension for 6-8 weeks postoperatively. During this time, patients were allowed to begin strengthening exercise of quadriceps and partial weightbearing with knee immobilizer. After removal of the knee immobilizer, patients were instructed to begin to regain knee range of motion followed by gradual physiotherapy. At 3 months postoperatively, full weightbearing was allowed and full range of motion was encouraged as tolerated.

### Outcome measurements

Clinical outcomes including knee range of motion (ROM), 36-Item Short-Form Health survey (SF-36) scores, Knee Injury and Osteoarthritis Outcome Scores (KOOS), and Kujala scores were collected and analyzed for all enrolled patients. These functional scores, ROM, and peak torque were measured at pre-operation and final follow-up visit.

### SF-36 score

The SF-36 scores, consisting of the physical component score (PCS) and mental component score (MCS), is a well-established method in assessing outcomes in diverse patient population and has been widely used for assessing the impact of traumatic injuries on healthy-related quality of life [14, 15]. The higher the score, the better the health status.

### Knee Injury and Osteoarthritis Outcome Score

The KOOS scores, including five subscales: pain, symptoms, activities of daily living (ADL), function in sport and recreation, and knee-related quality of life (QOL), is regarded as a method to assess knee injury that can lead to post-traumatic osteoarthritis [16, 17]. A normalized score (0 score indicating extreme symptoms and 100 scores indicating no symptoms).

### Kujala scoring system

The Kujala scores, consisting of 13 subscales with a score ranging from 5 to 10 points each subscale, are widely used to evaluate subjective symptoms and functional limitation of patellofemoral disorders [18]. A normalized score (0 score indicating extreme symptoms and 100 scores indicating normal conditions).

### Biodex System dynamometer

Biodex System dynamometer was used to evaluate quadriceps femoris muscle power following measurement of peak torque, which has been demonstrated to be a well-validated instrument [19].

### Complications

Complications regarding the two surgical techniques included calcification, infection, nonunion, implant failure, soft tissue irritation, and the development of patellofemoral arthritis. For each patient, the complications within the follow-up period were recorded and compared.

### Statistical analysis

SPSS software (version 25.0, IBM Corp., USA) was performed for statistical analysis. Continuous variables were presented as mean ± standard deviation. Categorical variables, presented as numbers or percentages, were compared by chi-square test. An Independent sample *t* test was performed for statistical analysis of clinical outcomes between the ORIF group and total patellectomy group. *p* value<0.05 was regarded statistically significant for all tests.

## Results

### Follow-up

All included patients were follow-up pre-operative and at the final follow-up. Functional scores, including SF-36 score, KOOS score, Kujala score, and range of motion were recorded to evaluate patients’ postoperative functional recovery. Clinical examination in measuring peak torque with Biodex System dynamometer to quantitatively evaluate the recovery of quadriceps femoris muscle power. The average follow-up periods of the total patellectomy and ORIF group were 17.2±5.6 and 16.8±4.9 years, and no significant difference was found between the two groups (*p*>0.05).

### General results

Between January 1987 and December 2003, the patient demographic characteristics of the 35 patients enrolled in this study are summarized in Table 1. Among the 35 patients, there were 17 patients in the patellectomy (15 males and 2 females) and 18 patients in the ORIF group (14 males and 4 females), and there were no statistically significant differences between the two groups with regard to age ([50.6±17.1] years *vs* [52.1±16.3] years, *p*=0.792), sex (male/female, [15/2] *vs* [14/4], *p*=0.631), side (left/right, [10/7] *vs* [12/6], *p*=0.631), time interval from injury to surgery (3.6±2.7 *vs* 4.1±3.5 days, *p*=0.545), and Regazzoni classification (type C2/C3, [3/14] *vs* [2/16], *p*=0.581).

In the total patellectomy group, eight patients resulted from a traffic accident, six patients resulted from a fall from height, and three patients resulted from a direct blow. In the ORIF group, nine patients resulted from a traffic accident, five patients resulted from a fall from height, and four patients resulted from a direct blow. Also, no significant difference was found regarding the type of trauma between the two groups (traffic accident/fall from height/direct blow, [8/6/3] *vs* [9/5/4], *p*=0.876).

### Clinical outcomes

The patients’ knee functional scores were recorded and analyzed, as shown in Table 2. The *range of motion* (ROM) in the total patellectomy was similar to the ORIF group [injured knee: 120.4±3.1° *vs* 118.6±3.3°; uninjured knee: 126.5±2.8° *vs* 127.3±1.7°], showing no statistically significant difference between the two groups (*p*>0.05). In addition, no statistically significant difference was identified in the peak torque [injured knee: total patellectomy 96.2±2.3 *vs* ORIF group 97.3±2.6, *p*>0.05; uninjured knee: total patellectomy 107.6±2.1 *vs* ORIF group 106.3±1.8, *p*>0.05].

**Table 2:** Comparison of clinical outcomes between groups

Variables	Total patellectomy group (*n*=17)	ORIF group (*n*=18)	*p* value
Operation time (minute)	47.5±12.1	68.8±22.3	0.001*
Range of motion (°)			
Injured knee	120.4±3.1	118.6±3.3	0.106
Uninjured knee	126.5±2.8	127.3±1.7	0.311
Peak torque (N· m)			
Injured knee	96.2±2.3	97.3±2.6	0.195
Uninjured knee	107.6±2.1	106.3±1.8	0.057
SF-36 score			
PCS	64.1±18.0	61.5±17.9	0.676
MCS	55.1±13.8	54.3±12.4	0.858
KOOS score	76.3±12.1	73.4±11.7	0.473
Kujala score	67.6±11.8	70.8±11.9	0.440

*ORIF* open reduction and internal fixation, *PCS* physical component score, *MCS* mental component score, *KOOS* Knee Injury and Osteoarthritis Outcome Score

The values are presented as the mean±SD

*Statistically significant difference between the two groups (*P*<0.05)

There were statistically significant differences in the postoperative SF-36 score between the two groups. The mean PCS score and MCS score in the total patellectomy were higher than the ORIF group, while no statistically significant difference was observed between the two groups (64.1±18.0 *vs* 61.5±17.9; 55.1±13.8 *vs* 54.3±12.4, both *p*>0.05). Furthermore, there were statistically significant differences in the postoperative KOOS score between the two groups. The mean KOOS score in the total patellectomy was higher than the ORIF group, while no statistically significant difference was observed between the two groups (76.3±12.1 *vs* 73.4±11.7, *p*>0.05). Similarly, there were statistically significant differences in the postoperative Kujala scores between the two groups. The mean Kujala score in the total patellectomy was lower than the ORIF group, while no statistically significant difference was observed between the two groups (67.6±11.8 *vs* 70.8±11.9, *p*>0.05).

### Complications

**Fig. 8:**
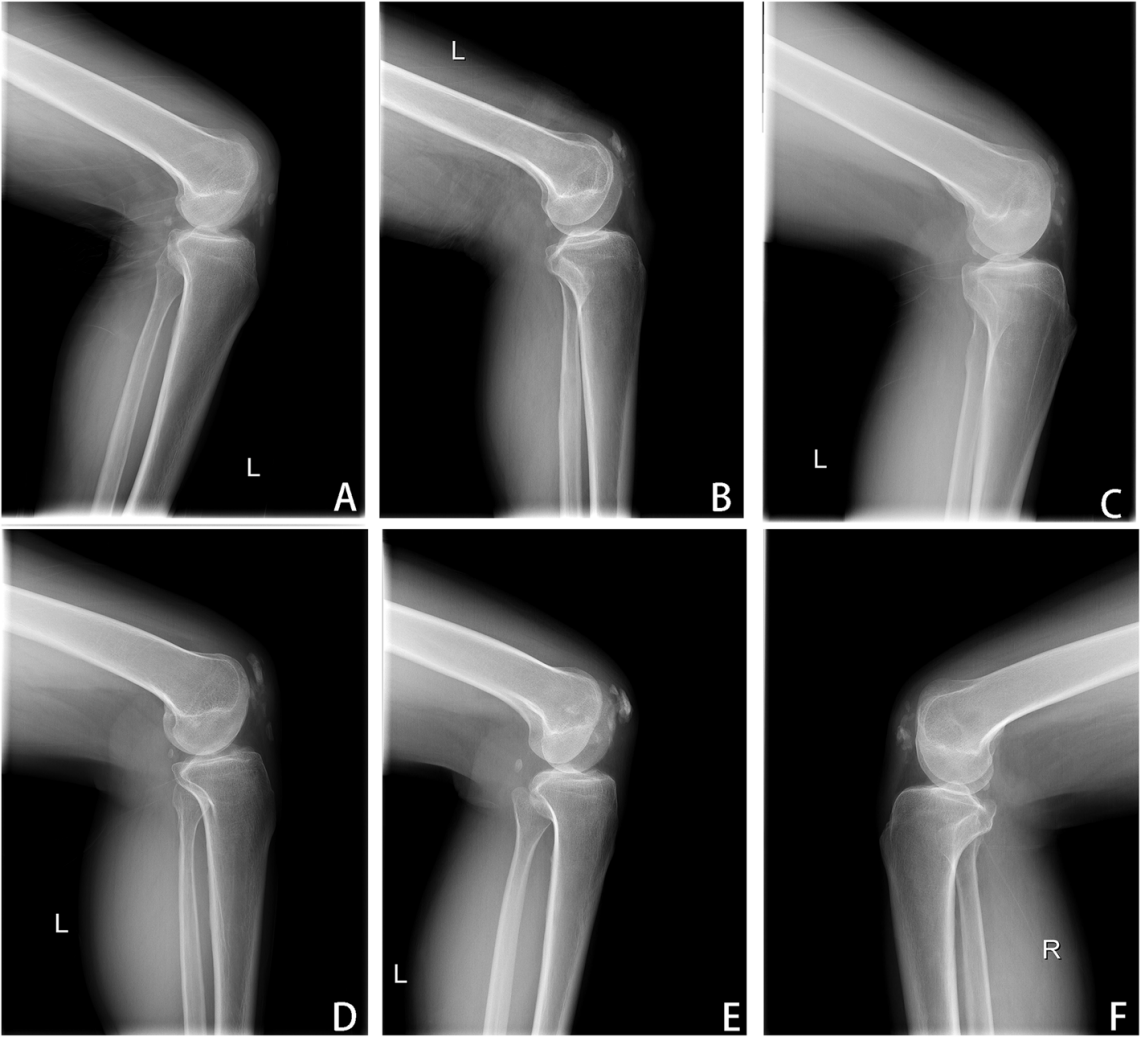
Postoperative lateral knee radiographs showed patients developed calcification after total patellectomy at the final follow-up

Postoperative complications related to the different surgical techniques were presented in Table 3. In the total patellectomy group, six patients (6 of 17, 35.3%) had calcification (Fig. 8) and did not require additional surgical intervention. However, there were twelve complications in the ORIF group with tension band wiring, of which one patient had fracture nonunion and four patients had implant-associated problems, including two patients of implant failure and two patients of soft tissue irritation. Patients with a complaint of soft tissue irritation had removed the fixation earlier. In addition, there were another two patients who developed superficial wound infection and performed irrigation and debridement; Of note, the most common complication in the ORIF group was the degenerative changes of the patellofemoral joint, accounting for 27.8% (5 of 18 patients) of all patella fractures that treated by tension banding wiring.

**Table 3:** Comparison of postoperative complications between groups

Technique	*n*	Calcification *n* (%)	Infection *n* (%)	Nonunion *n* (%)	Implant failure *n* (%)	Soft tissue irritation *n* (%)	Patellofemoral arthritis *n* (%)
TP	17	6 (35.3%)	-	-	-	-	-
ORIF	18	-	2 (11.1%)	1 (5.6%)	2 (11.1%)	2 (11.1%)	5 (27.8%)
Total	35	6 (17.1%)	2 (5.7%)	1 (2.9%)	2 (5.7%)	2 (5.7%)	5 (14.3%)

*TP* total patellectomy, *ORIF* open reduction and internal fixation

## Discussion

Orthopedic surgeons considered that the patella fracture fragments should be preserved as much as possible due to potential detrimental effect on quadriceps power, and total patellectomy is viewed as the last resort in treating patients with highly comminuted patellar fractures. In the present study, we compared two surgical techniques that were performed at our institute in treating these fractures, specifically, total patellectomy and internal fixation with tension band wiring. However, in contrast to traditional viewpoints, the present study demonstrated that total patellectomy can also achieve acceptable and satisfactory clinical outcomes with a minimum of 10 years follow-up as compared with the tension band wiring. Despite total patellectomy developed higher rates of calcifications, they also showed favorable functional scores and an acceptable range of motion.

Even though the significant advances in fixation techniques, including titanium-nickel alloy [20], titanium cable [21], angle plate fixation [22], and low profile mesh plate [23]; however, it remains challenging in the management of highly comminuted patella fractures as they are often associated with small multi-fragmentary fractures. Furthermore, the complexity of patella comminuted fractures often hinders accurate reduction of the articular surface, therefore, total patellectomy is sometimes inevitable [9]. However, the total patellectomy procedure, which includes resection of fracture fragments and reconstruction quadriceps femoris tendon and patellar tendon, is a controversial surgical technique for comminuted fractures.

Despite some published studies showed that unsatisfactory clinical outcomes in patellectomy, and they concluded that this technique destroyed the patella leverage function in extensor mechanism. Our results reported the favorable clinical outcomes of utilizing the total patellectomy technique for highly comminuted patellar fractures on the longest follow-up time reported thus far, and it is the first study to compare it with the traditional fixation technique with tension band wiring. Of note, total patellectomy had a significantly shorter operation time than the ORIF group (47.5±12.1 min compared to 68.8±22.3 min, respectively, *p*<0.05). In addition to shorter operation time, this present study also identified that patellectomy had comparable ROM of the knee joint, peak torque, and functional scores at the final follow-up when compared to tension band wiring according to the clinical evaluations

Although there are several different kinds of fixation techniques for patellar comminuted fractures, surgical treatment for highly comminuted patellar fractures involving the articular surface is still complicated and challenging for orthopedic surgeons, mainly owing to the fragments are typically small and comminuted, and fixation is more inclined to fail. Sometimes, it is difficult to maintain the stability and restore joint surface regularity in comminuted fractures. Notably, total patellectomy was not carried out indiscriminately, but performed only when precise indications were recognized. In this study, we just compared total patellectomy with tension band wiring regarding long-term clinical outcomes in treating highly patellar comminuted fractures, and we did not compare outcomes of total patellectomy with other osteosynthesis techniques directly. Our results suggested that total patellectomy may be an effective and reliable alternative treatment in patients with highly comminuted patella fractures when compared to tension band wiring. However, at present, numerous variations of the surgical techniques have been developed owing to the rapid evolution of internal fixation, anatomical reduction with novel internal fixation may have superior clinical outcomes than total patellectomy.

The rates of complication were relatively lower in the total patellectomy group as compared with the traditional fixation technique using tension banding wiring. Calcification was the most commonly observed complication (35.5%) following total patellectomy, most probably explained by the fact that persistent irritation of the quadriceps femoris tendon and the patellar tendon. However, the calcification did not interfere with the recovery of knee function. We concluded that the formation of calcification may be as a role acting as a substitute patella. On the other hand, patellofemoral arthritis was the most common complication (27.8%, 5 of 18 patients) with tension band wiring and they often complained of anterior knee pain when flexing. Mehdi et al. [24] showed that 8.5% (17 of 203 patients) of patellar fracture patients who were treated with tension band wiring developed patellofemoral arthritis. A possible reason for the relatively high rates of patellofemoral arthritis in the present study may be due to the included patients were highly comminuted patella fractures.

Despite preserving the patella and restoring the functional integrity of the extensor mechanism, our results demonstrated that these fractures treated by tension band wiring are often susceptible to degenerative changes in the patellofemoral joint at long-term follow-up. This condition may be due to the difficulty for an anatomical articular reduction in these fractures. Thus, the postoperative irregular contour of the patellar articular surface may lead to the progression of patellofemoral arthritis.

Although it demonstrated the potential advantages of total patellectomy in patellar comminuted fractures, this study also has several limitations. First, this study included a small number of patients, as patients were from a single center and the strict patient inclusion criteria. Second, internal fixation with tension band wiring is a relatively old surgical technique for comminuted patellar fractures, which might be a deficiency relative to the present improved fixation technique. Third, this retrospective study was conducted between 1987 and 2003, and we did not evaluate the patient’s motor function loss at the predetermined time points, which may have potentially increased bias. Fourth, with the rapid development of a variety of internal fixation devices and stabilization techniques are available for patellar comminuted fracture fixation, we have to admit that the evaluation of the functional outcome may differ significantly between the total patellectomy and other new fixation technologies. Future studies involving prospective and multicenter with a large number of patients are needed to further explore the difference between patellectomy and other novel surgical procedures on highly comminuted patella fractures.

Despite these limitations, however, this study evaluated the long-term clinical outcomes of total patellectomy, identifying that satisfactory clinical result and few complications can be achieved. Calcification was the most common complication with total patellectomy, most probably explained by the fact that persistent rubbing of the quadriceps femoris tendon on the condylar surface. In addition, we used the combination of subjective outcome scores and objective measurement tools to provide findings with long-term clinical outcomes.

## Conclusion

Total patellectomy is a good treatment option in treating highly comminuted patella fracture with favorable clinical outcomes at the long-term follow-up. In contrast to conventional opinions, our results suggested that total patellectomy may be an effective and reliable alternative treatment in patients with highly comminuted patella fractures when anatomically reduction was difficult. Even though total patellectomy caused increased rates of calcification, any restricted range of motion and patellofemoral pain was not developed.

## Data Availability

All the data and material involving this article will be available upon request by sending an e-mail to the first author.

## Abbreviations

ORIF: Open reduction and internal fixation
PCS: Physical component score
MCS: Mental component score
KOOS: Knee Injury and Osteoarthritis Outcome Score
ROM: Range of motion
SF-36: Short-Form Health Survey
ADL: Activities of daily living
QOL: Quality of life
